# Validation of miRNA signatures for ovarian cancer earlier detection in the pre-diagnosis setting using machine learning approaches

**DOI:** 10.3389/fonc.2024.1389066

**Published:** 2024-06-25

**Authors:** Konrad Stawiski, Renée T. Fortner, Luca Pestarino, Sinan U. Umu, Rudolf Kaaks, Trine B. Rounge, Kevin M. Elias, Wojciech Fendler, Hilde Langseth

**Affiliations:** ^1^ Department of Biostatistics and Translational Medicine, Medical University of Lodz, Lodz, Poland; ^2^ Department of Research, Cancer Registry of Norway, Norwegian Institute of Public Health, Oslo, Norway; ^3^ Division of Cancer Epidemiology, German Cancer Research Center, Heidelberg, Germany; ^4^ Department of Gynecological Oncology, Division of Cancer Medicine, Oslo University Hospital, Oslo, Norway; ^5^ Centre for Bioinformatics, Department of Pharmacy, University of Oslo, Oslo, Norway; ^6^ Division of Gynecologic Oncology, Department of Obstetrics, Gynecology and Reproductive Biology, Brigham and Women’s Hospital, Boston, MA, United States; ^7^ Harvard Medical School, Boston, MA, United States; ^8^ Department of Radiation Oncology, Dana-Farber Cancer Institute, Boston, MA, United States; ^9^ Department of Epidemiology and Biostatistics, School of Public Health, Imperial College London, London, United Kingdom

**Keywords:** ovarian cancer, microRNAs, early detection, machine learning, sequencing

## Abstract

**Introduction:**

Effective strategies for early detection of epithelial ovarian cancer are lacking. We evaluated whether a panel of 14 previously established circulating microRNAs could discriminate between cases diagnosed <2 years after serum collection and those diagnosed 2–7 years after serum collection. miRNA sequencing data from subsequent ovarian cancer cases were obtained as part of the ongoing multi-cancer JanusRNA project, utilizing pre-diagnostic serum samples from the Janus Serum Bank and linked to the Cancer Registry of Norway for cancer outcomes.

**Methods:**

We included a total of 80 ovarian cancer cases contributing 80 serum samples and compared 40 serum samples from cases with samples collected <2 years prior to diagnosis with 40 serum samples from cases with sample collection ≥2 to 7 years. We employed the extreme gradient boosting (XGBoost) algorithm to train a binary classification model using 70% of the available data, while the model was tested on the remaining 30% of the dataset.

**Results:**

The performance of the model was evaluated using repeated holdout validation. The previously established set of miRNAs achieved a median area under the receiver operating characteristic curve (AUC) of 0.771 in the test sets. Four out of 14 miRNAs (hsa-miR-200a-3p, hsa-miR-1246, hsa-miR-203a-3p, hsa-miR-23b-3p) exhibited higher expression levels closer to diagnosis, consistent with the previously reported upregulation in cancer cases, with statistical significance observed only for hsa-miR-200a-3p (beta=0.14; p=0.04).

**Discussion:**

The discrimination potential of the selected models provides evidence of the robustness of the miRNA signature for ovarian cancer.

## Introduction

Epithelial ovarian cancer is the most lethal of the gynaecologic malignancies, with poor long-term survival (5-year relative survival of 40–45%) (1, 2). Effective strategies for early detection are lacking, and non-specific early symptoms of disease (e.g., bloating, feeling of fullness, frequent urination) (3, 4) contribute to delayed diagnosis. The most widely used blood-based biomarker for ovarian cancer early detection is CA125; however, screening with CA125 in combination with transvaginal ultrasound (TVUS) has not demonstrated a survival benefit in large screening trials (5, 6).

MicroRNAs (miRNAs) are short (18–24 nucleotide) non-coding RNAs that regulate post-transcriptional gene expression, and which have regulatory roles in a variety of cellular functions. More than 2,500 miRNAs have been identified in humans, in tissues, and in circulation in MiRBase (7). Besides their common intracellular localization, miRNAs are present and stable in all body fluids, including blood plasma and serum (8). Aberrant miRNA expression has been well characterized in cancer and other health conditions, and combination signatures of individual miRNAs in blood have been shown to be predictive for cancer risk and prognosis (9–13). Preliminary studies have suggested that circulating miRNA profiles are altered in women with ovarian cancer and distinct miRNA profiles have also been found to be associated with the prognosis of ovarian cancer patients (14, 15). A methodological limitation of many early detection marker discovery studies as of to date is that they have been based almost exclusively on comparisons between women already diagnosed with cancer and cancer-free controls. We have recently identified and validated a panel of miRNAs using next generation sequencing and neural network statistical models (16). This model was developed and validated in independent study populations, and distinguishes between prevalent invasive ovarian cancers and healthy women with overall sensitivity of 75% at 100% specificity and with significantly better performance than CA125 alone; further, the identified panel was specific to ovarian cancer, with no predictive power for other types of cancers, and was not correlated with circulating CA125 concentrations (16).

The aim of the current study was to evaluate the feasibility of applying previously defined miRNA signatures from retrospective case-control studies in an independent study with miRNA sequencing data from samples collected prior to ovarian cancer diagnosis, and to evaluate the performance of these signatures in these pre-diagnosis samples.

## Materials and methods

Small RNA sequencing data was generated as part of the large, ongoing multi-cancer JanusRNA project (17) at the Cancer Registry of Norway (CRN). JanusRNA has its origin in the large population-based Janus Serum Bank Cohort (JSB) established in 1973 and is a population-based prospective cancer biobank including 318,628 participants with serum samples collected between 1972–2004, as previously described (18, 19). Incident cancer cases are identified by linking the JSB to CRN, which was established in 1951 and includes mandatory reported information on all new cancer cases in Norway (20).

### Study design and selection of participants from the JanusRNA study

**Figure 1:**
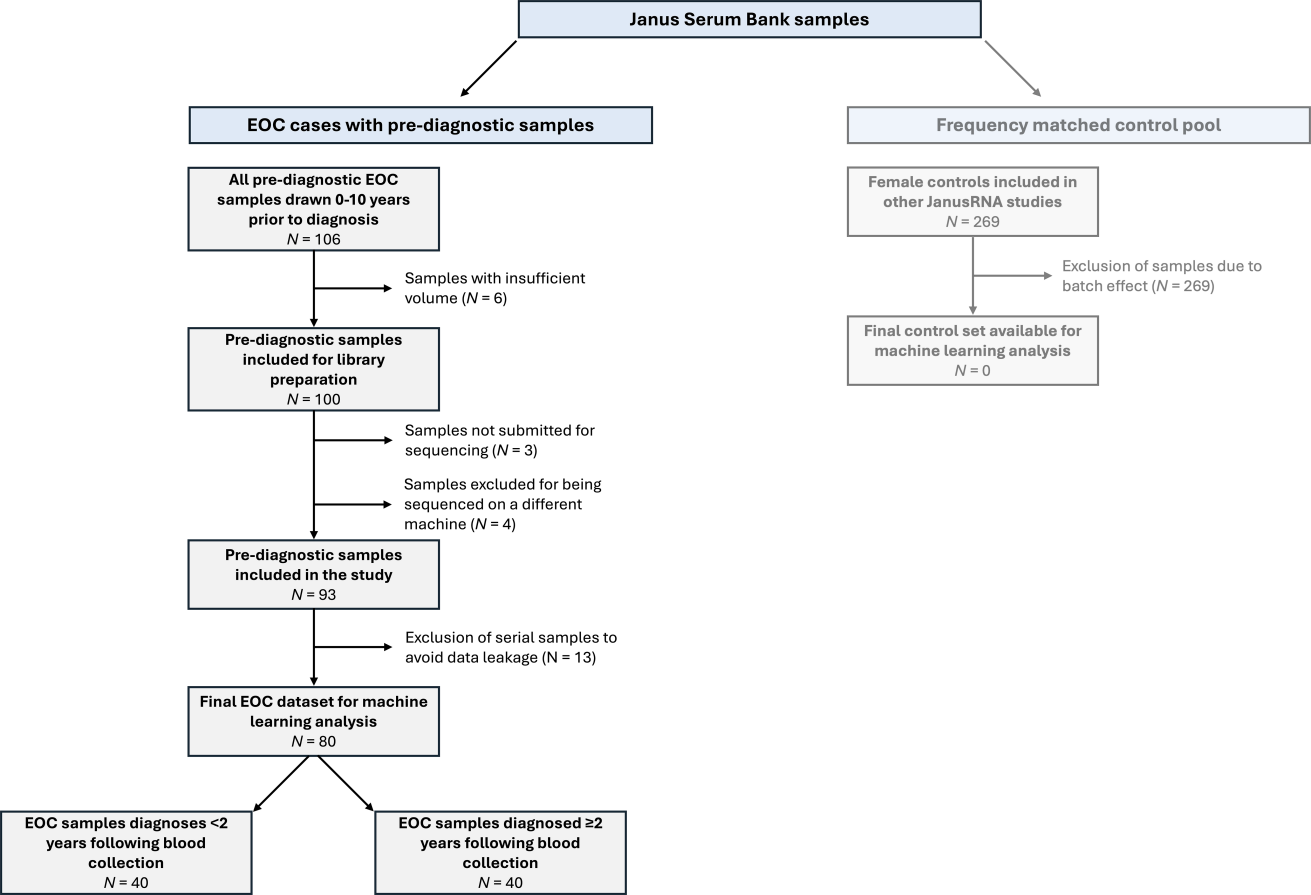
Samples included in the analysis and exclusion criteria (EOC, epithelial ovarian cancer).

The overall design in JanusRNA is restricted to cases who donated at least one blood sample up to 10 years prior to a diagnosis, and a frequency matched control group that remained cancer-free 10 years after blood donation. This resulted in the inclusion of 80 invasive ovarian cancer cases with a total of 106 samples (n=14 with two samples and n=4 with three samples or more) diagnosed up to 7 years following blood collection. A common control set for all cancers included in JanusRNA were measured together with other cancer types in the JanusRNA study (17, 21). Due to a moderate batch effect between ovarian cases and controls discovered using principal components analysis (**Supplementary Figure 1**), controls were discarded in this study. Thus, an alternative approach was adopted in which we compared samples collected close to diagnosis to those further from diagnosis. A detailed description of the data set used, and the samples exclusion criteria is shown in **Figure 1**.

This study was conducted as part of the ongoing work of the PREDICT consortium (Prospective Early Detection Consortium for Ovarian Cancer), which has a specific focus on early detection biomarkers for ovarian cancer identified in samples proximate to diagnosis (i.e., <1.5–2 years). Thus, the cases from JanusRNA diagnosed <2 years following blood collection were of primary interest for potential earlier detection and were included in a subgroup of cases diagnosed proximate to blood collection, with the cases with ≥2 years between blood sampling and diagnosis as the comparison group with samples donated more distant from blood collection. The selection of cases diagnosed <2 years after blood collection is in line with the point at which discrimination is observed between cases and controls in prior studies of protein-based biomarkers (22). To avoid the possibility of any data leakage between the training and test set samples, we selected only a single serum sample for each individual. The samples for cases diagnosed <2 years following blood collection were matched to the samples for cases diagnosed ≥2 years following blood collection according to age and blood donor group using the optmatch package (v0.9–13) in R (v4.0.0). Blood donor group is a technical confounder which combines the effect of sample treatment at donation and storage time (23). After the matching was completed, a total of 80 samples (n=40 diagnosed <2 years following blood draw, n=40 diagnosed ≥2 years following blood draw) were used for the analyses.

### Laboratory processing

RNA was extracted from 2 x 200 ml serum using phenol chloroform phase separation and the miRNeasy Serum/Plasma kit (Cat. no 1071073, Qiagen) on a QIAcube (Qiagen). Glycogen (Cat. no AM9510, Invitrogen) was used as a carrier during the RNA extraction step. Small RNA-seq was performed using NEBNext^®^ Small RNA Library Prep Set for Illumina (Cat. No E7300, New England Biolabs Inc.). We performed size selection using a 3% Agarose Gel Cassette (Cat. No CSD3010) on a Pippin Prep (Sage Science) with a cut size optimized to cover RNA molecules from 17 to 47 nt in length. Sequencing libraries were indexed and 12 samples were sequenced per lane of a HiSeq 2500 (Illumina). The average depth was 18M reads per sample. Our small RNA sequencing protocol has been previously described (21).

### Bioinformatics analyses

Raw reads were processed with the “sncRNA-workflow” pipeline (v1.0.0) (21). This pipeline includes different steps, such as quality control, adapter trimming, read mapping, read counting and the creation of count tables. The mapping was performed with mature miRNA reference from miRBase (v22.1) (7). AdapterRemoval was used for adapter trimming (24), whilst Bowtie2 (25) was employed for mapping reads to the human genome (hg38) with an average mapping ratio of 70%. miRNA annotation was performed using the SeqBuster tool. The miRNA counts were then normalised using a read per million (RPM) normalization (26).

### miRNA set selection

We evaluated two sets of miRNAs for validation based on our previous publication (16). The miRNAs were identified using feature selection by expression fold change and a miRNA algorithm was derived using a neural network algorithm, using sequencing data; the miRNAs and algorithm were subsequently validated using qPCR. The original sequencing files were downloaded from GEO (https://www.ncbi.nlm.nih.gov/geo/query/acc.cgi?acc=GSE94533) and processed using the same pipeline as described in the previous sections. The primary set was acquired from Elias et al. (16), and included 14 miRNAs: *miR-23b-3p, miR-29a-3p, miR-32–5p, miR-92a-3p, miR-150–5p, miR-200a-3p, miR-200c-3p, miR-203a-3p, miR-320c, miR-320d, miR-335–5p, miR-450b-5p, miR-1246 and miR-1307–5p.* This signature has been reported to perform well regardless of disease histotype and stage (16). The second set used on Keller et al. (27), included the same miRNAs as previously listed with the exception of *miR-1307–3p* replacing *miR-1307–5p*; this set was evaluated in a secondary analysis.

### Model development

The extreme gradient boosting (XGBoost) algorithm was used to train binary classification models using the pre-defined 14 miRNAs. Due to significant technological differences in the RNA profiles between studies (**Supplementary Figure 2**), the original model [as developed in Elias et al. (16)] could not be directly reapplied to the JanusRNA dataset. XGBoost is a scalable tree boosting model development method that uses regularization to handle overfitting and high dimensionality. Previous research has suggested that XGBoost outperforms complicated methods like deep learning on tabular data (28). It can also capture non-linear relationships (29). We utilized the R (v4.0.0) implementation of this algorithm, XGBoost (v1.5.2.1). We randomly separated the dataset into training (70%) and test (30%) sets (hold-out validation). The model performance was evaluated on the test datasets using the area under the ROC curve (AUC) value. We repeated the creation of training and test sets 5 times with different seed values and calculated AUC values. For each repetition, the best value of the *subsample* hyperparameter was selected using a grid search approach combined with a five-fold cross-validation on the training set. The other hyperparameters were set according to the results of our previous work (30) after having considered the similar distribution of the two data sets. By reporting the median AUC in the test set, we were able to validate the miRNA sets and evaluate the presence of overfitting. Feature importance values were exported using the *xgb.importance* function of the XGBoost package. Then, we calculated average importance values to sort the final list of miRNA biomarkers.

Finally, as an additional step in miRNA validation, we conducted a negative control experiment by randomly generating five sets of 14 miRNAs (no filtering was applied for the selection). We then evaluated the performance of these randomly selected miRNA sets using our model development workflow, as described above. This allowed us to establish a background comparison and compare the performance of the randomly selected miRNAs with the performance of the *a priori* defined miRNA sets. We evaluated Spearman correlations between the *a priori* selected miRNAs and the randomly selected miRNAs given expected correlations between individual miRNAs.

### Linear trend assessment

Lastly, we checked if changes in selected miRNAs present dynamics concordant with changes observed for comparison between ovarian cancer cases and controls. Using univariable linear regression, we estimated linear trends between normalized miRNA expression and time to diagnosis. We calculated beta coefficients and p-values. Beta coefficients greater than 0 indicated that miRNA abundance was rising closer to diagnosis. The range between -0.1 and 0.1 indicated no association. Those results were compared to log2-transformed fold-changes (log2FC) obtained on whole dataset in Elias et al. (16) using OmicSelector package (26).

## Results

**Figure 2:**
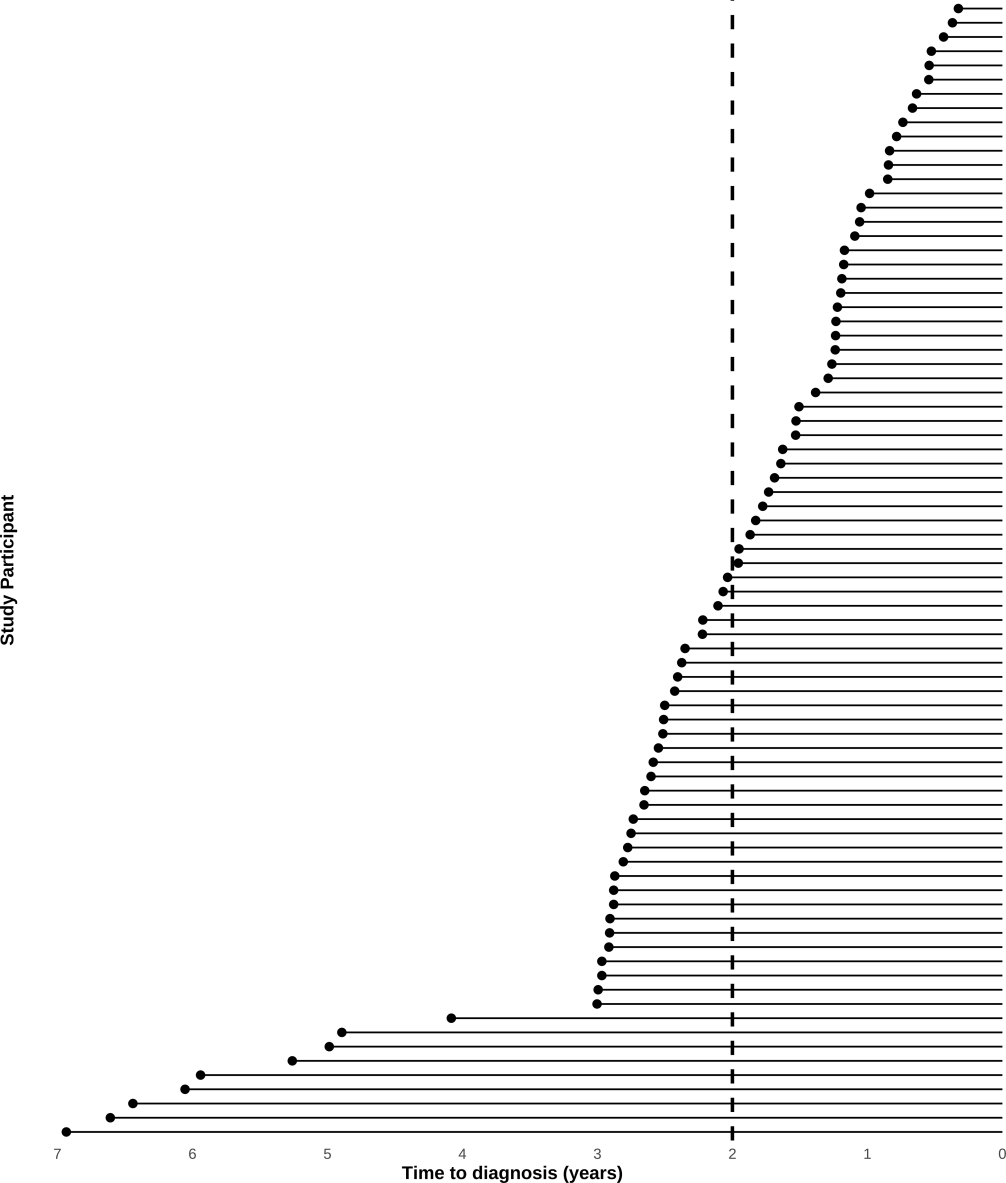
Timing of sample collection by time between blood collection and diagnosis. We used 2 years prior to diagnosis as a threshold for the comparison groups, which were later used in binary classification models for validation and discovery.

Mean age at diagnosis was 48.9 years in the cases diagnosed <2 years following blood collection and 48.0 years in the cases diagnosed ≥2 years following blood collection. Tumor histology was generally similar in both groups, though with a higher proportion of carcinomas, not otherwise specified (NOS) among cases diagnosed more distant from blood collection (58% vs. 50%), and a somewhat higher proportion of endometrioid tumors in the cases diagnosed more proximate to blood collection. **Figure 2** shows the timing of blood sample collection and diagnosis in the study sample. The mean (SD) of years between blood collection and diagnosis was 1.16 (0.46) years for early-case samples and 3.31 (1.39) years for late-case samples (**Table 1**).

**Table 1:** Baseline characteristics [n (%) or mean (standard deviation)] of the study participants contributing serum samples and diagnosed with invasive epithelial ovarian cancer in the windows 0 to <2 years and ≥2 to 7 years after blood collection.

	Time between blood collection and diagnosis		
	0 to <2 years	2 to 7 years	P-value
Number of samples	40	40	
Tumor histology			0.16
Serous	4 (10%)	4 (10%)	
Endometrioid	8 (20%)	5 (12.5%)	
Mucinous	6 (15%)	6 (15%)	
Carcinoma, NOS	20 (50%)	23 (57.5%)	
Clear cell	2 (5%)	2 (5%)	
Age at blood sampling, years	48.16(9.67)	45.23 (7.33)	0.13
Age at diagnosis, years	48.88 (9.57)	48.02(7.72)	0.66
Prediagnostic sampling time, years	1.16 (0.46)	3.31 (1.39)	<0.01
Disease stage			0.23
Localized/regional	19 (47.5%)	19 (47.5%)	
Distant metastases	21 (52.5%)	21 (52.5%)	

### Previously derived miRNA signature discriminates between cases diagnosed proximate to and distant from blood collection in JanusRNA

**Figure 3:**
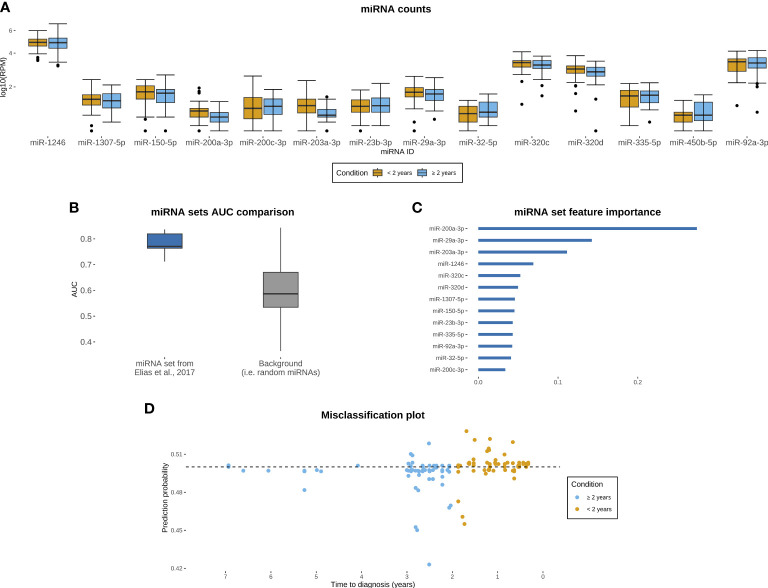
Evaluation of miRNA set from dataset described in Elias et al. (16) in JanusRNA ovarian cancer case set. **(A)** miRNA counts for the evaluated panel, by time between blood collection and diagnosis (<2 years, ≥2 to 7 years; microRNAs log10 transformed for visualization and zero values omitted from the plot); **(B)** Boxplots of model performance for the primary evaluated miRNA signature and random miRNA set; **(C)** Feature importance for each of the included miRNAs **(D)** Misclassification plot of the test samples based on the miRNA signature, y axis represents prediction probability for a given observation to have been sampled within 2 years prior to diagnosis.

Counts differed substantially across the evaluated miRNAs (**Figure 3A**). The miRNA signatures had median AUCs of 0.771 (IQR: 0.056) with an average of 0.781 (95% CI: 0.72, 0.84) for the miRNA signature from the Elias et al. (16) dataset (**Figure 3B**) and 0.753 (IQR: 0.076) with an average of 0.728 (95% CI: 0.64, 0.82) for the miRNA signature previously evaluated in the Keller et al. (27) dataset (**Supplementary Figure 3**) in the test datasets. The ROC curves for each of the five train/test split repetitions in the two miRNA sets are shown in **Supplementary Figure 4**. Randomly generated miRNAs had a median AUC of 0.587 (IQR: 0.135) and an average AUC of 0.6 (95% CI: 0.55, 0.65) (**Figure 3B**; 5 sets of 14 miRNAs shown in **Supplementary Figure 5**) The correlations between *a priori* selected and randomly selected miRNAs (**Supplementary Figure 6**) explain the above the average performances of the randomly generated miRNAs. When we investigated the feature importance of the *a priori* selected miRNA set models, we found that miR-200a-3p was the most important miRNAs for both sets followed by miR-29a-3p, mir-203a-3p and miR-1246 (**Figure 3C**). The miRNA that differed between the two sets was ranked 7^th^ in feature importance in the primary set (miR-1307–5p) and 4^th^ in the secondary set (miR-1307–3p). The misclassification plot (**Figure 3D**) shows the prediction probabilities of ovarian cancer diagnosed within the 2 years subsequent to blood collection in the five test sets using the primary miRNA signature. Although all the probabilities are included within the 0.42–0.53 range, the majority of those over 0.5 were assigned to the ovarian cancer cases diagnosed within 2 years of blood collection, demonstrating the prediction performance of the model.

Lastly, we examined the concordance between up- and downregulation in miRNAs observed in ovarian cancer cases and controls in Elias et al., compared to the linear trend we observed over time prior to diagnosis (**Table 2**; **Supplementary Figure 7**). We found that no temporal trend was observed for 10 out of 14 miRNAs. In the remaining miRNA, the trend was consistent with the change reported in the study by Elias et al. This miRNA exhibited an increase in expression over time and was also associated with upregulation in the original comparison of ovarian cancer cases and controls. However, among these miRNAs, only miR-200a-3p demonstrated a statistically significant observed trend.

**Table 2:** Temporal linear trends in selected miRNAs prior to ovarian cancer diagnosis compared to differential expression results observed in comparison between ovarian cancer cases and controls in original paper by Elias et al. (eLife 2017).

miRNA	Elias et al.		Our study		
	log2FC	p-value	beta	p-value	Interpretation
miR-200c-3p	1.56	0.000	0.08	0.27	No change over time
miR-320d	1.06	0.000	0.01	0.88	No change over time
miR-320c	0.79	0.000	0.02	0.78	No change over time
miR-200a-3p	1.03	0.000	0.15	0.04	Concordant and significant trend
miR-1307–5p	0.50	0.000	-0.04	0.59	No change over time
miR-1246	1.10	0.001	0.13	0.08	Concordant and non-significant trend
miR-203a-3p	1.05	0.001	0.12	0.11	Concordant and non-significant trend
miR-450b-5p	0.87	0.002	0.06	0.44	No change over time
miR-23b-3p	0.60	0.003	0.14	0.07	Concordant and non-significant trend
miR-32–5p	0.56	0.004	0.08	0.32	No change over time
miR-335–5p	0.48	0.004	-0.02	0.80	No change over time
miR-29a-3p	0.48	0.011	0.05	0.50	No change over time
miR-92a-3p	-0.21	0.126	-0.03	0.71	No change over time
miR-150–5p	0.04	0.883	-0.01	0.91	No change over time

miRNAs were considered concordant if they exhibited similar increase/decrease over time prior to diagnosis (linear regression beta coefficient and p-value) when compared to up-/downregulation (log2 fold-change; log2FC) reported in the Elias paper. Beta coefficients close to 0 (-0.1 < beta < 0.1) were interpreted as a lack of temporal trend.

## Discussion

In this study we evaluated sets of miRNAs previously identified as potential early detection markers in prevalent ovarian cancer cases and controls in an independent study population with serum samples collected prior to diagnosis, demonstrating that these miRNA sets discriminated between cases diagnosed in the relative short-term following blood collection (<2 years) and those diagnosed later (≥2–7 years). The obtained median AUC metrics on the test set exceeded the threshold of 0.75, which is recognized as a criterion for potential clinical usability (31). This indicates that significant and discriminatory changes occurred in the selected miRNA sets over time as ovarian cancer developed. The consistent performance of these miRNAs suggests that they form a robust set worthy of further evaluation and validation.

This provides support for the potential of these miRNAs for ovarian cancer early detection, though further studies are required. We compared cases diagnosed proximate to blood collection against those diagnosed more distant from blood collection for this study. Ideally, a representative cancer-free control group would have been used for comparison, as any early signal of a developing neoplasm more than 2 years prior to diagnosis would interfere with the performance of the miRNA sets in the current setting. Further, the performance of the *a priori* defined miRNA signature was observed in the context of limited comparability of the sequencing methodology between our study and the study in which the signature was derived; this was a limitation of this study. Despite extensive effort to unify the approach for post-sequencing analysis, marked heterogeneity in the data was noted across the sequencing profiles, suggesting inherent differences in sequencing technologies that made a direct transfer of models impossible between the two datasets. Our observation that the 5 sets of 14 randomly selected miRNAs all had AUCs >0.50 is in line with the correlations between individual miRNAs within the *a priori* selected and randomly select sets.

Our study revealed that among 14 miRNAs analyzed, miRNA-200a-3p was the only miRNA demonstrating a significant difference in expression in samples collected more distant from diagnosis, as compared to those collected proximate to diagnosis. While miRNA-200a has been commonly linked to oncogenesis (32), our research is pioneering in evaluating trends by timing of serum collection in the time window prior to ovarian cancer diagnosis. Beta coefficients, although not statistically significant, suggested similar trends for miR-1246, miR-203a-3p, and miR-23b-3p, i.e. demonstrated increased expression levels as the time to diagnosis decreased. The dynamics of these miRNAs in circulation prior to ovarian cancer diagnosis merit further investigation in larger studies, and evaluations of differences in miRNA patterns in pre-diagnosis samples from individuals with ovarian cancer and cancer-free controls are needed.

The relative stability of the remaining miRNAs could suggest that either no relationship with cancer stage exists or that the observed miRNA profile is associated with an increased risk of ovarian cancer development. Our recent work suggests that the miRNA profile could be associated with states linked to an increased risk of cancer development, such as BRCA1/2 mutation (33) or exposure to risk factors like ionizing radiation (34). The discovery of miRNA-related states of increased cancer risk may be crucial for early detection and possible screening in high-risk populations.

Beyond the findings on the miRNA signature, we have reached two principal conclusions regarding our applied approach. First, our initial aim was to perform validation of a previously developed model. However, significant batch effects and technological differences have rendered this validation impossible. Validating published models requires aligning technologies from the point of data generation and implementing normalization methods that are suitable for per-sample analysis and do not rely solely on batch effect correction. Unfortunately, currently available bioinformatic tools were unable to adequately address the substantial differences in the sequencing data in an unsupervised manner. To achieve validation, it will be necessary to generate a larger common dataset using a single platform, to which each group can apply various modelling techniques. Secondly, the miRNA set that was previously identified appears to possess predictive capacity. However, in order to calibrate the model for a reproducible test, it is crucial that all data originates from a common data generation stream. To address batch effects in decentralized scenarios, rigorous quality controls and adherence to *a priori* set normalization, filtering, and modeling standards will be necessary during the testing phase. Both topics are addressed in our ongoing research activities. Additionally, the creation of a larger common dataset and strict adherence to quality controls and standardized procedures are essential for reliable results in decentralized scenarios.

Taken together, these findings using existing sequencing data suggest that the evaluated miRNA sets previously identified for ovarian cancer early detection are relatively robust in discriminating between cases diagnosed proximate to blood collection from those diagnosed more distant from blood collection. A unified approach evaluating targeted panels of miRNAs and investigating performance in pre-diagnosis samples is required to advance these markers toward clinical utility.

## Data availability statement

The dataset used for this study data will be made available at Federated EGA (European Genomephenome Archive) Norway service after application to the study data access committee.

## Ethics statement

The studies involving humans were approved by Regional Ethics Committee South East. The studies were conducted in accordance with the local legislation and institutional requirements. The participants provided their written informed consent to participate in this study. No animal studies are presented in this manuscript. No potentially identifiable images or data are presented in this study.

## Author contributions

KS: Investigation, Formal analysis, Methodology, Software, Writing – review & editing. RF: Funding acquisition, Investigation, Supervision, Writing – original draft. LP: Data curation, Formal analysis, Investigation, Visualization, Writing – review & editing. SU: Formal analysis, Investigation, Methodology, Writing – review & editing. RK: Conceptualization, Funding acquisition, Investigation, Project administration, Writing – review & editing. TR: Conceptualization, Data curation, Funding acquisition, Investigation, Methodology, Software, Supervision, Writing – review & editing. KE: Conceptualization, Investigation, Methodology, Writing – review & editing. WF: Conceptualization, Investigation, Methodology, Supervision, Writing – review & editing. HL: Conceptualization, Funding acquisition, Investigation, Project administration, Supervision, Writing – review & editing.
