# Supplementary material for: Validation of miRNA signatures for ovarian cancer earlier detection in the pre-diagnosis setting using machine learning approaches
Source: Front Oncol. 2024 Jun 25;14:1389066. doi: 10.3389/fonc.2024.1389066 (PMC11231195; doi:10.3389/fonc.2024.1389066)
Supplement: Supplementary file 1 [file Image_1.pdf]

Supplementary Material

Validation of miRNA signatures for ovarian cancer earlier detection in the pre-diagnosis setting using machine learning approaches

Konrad Stawiski[1]*, Renée T. Fortner[2,3]*, Luca Pestarino[2,4*], Sinan U Umu[2], Rudolf Kaaks[3], Trine B. Rounge[2,5], Kevin M. Elias[6,7], Wojciech Fendler[1,8], Hilde Langseth[2,9**]**

*contributed equally and should be considered co-first authors
**contributed equally and should be considered co-last authors

[1]Department of Biostatistics and Translational Medicine, Medical University of Lodz, Lodz, Poland

[2]Department of Research, Cancer Registry of Norway, Norwegian Institute of Public Health, Oslo, Norway

[3]Division of Cancer Epidemiology, German Cancer Research Center, Heidelberg, Germany

[4]Department of Gynecological Oncology, Division of Cancer Medicine, Oslo University Hospital, Oslo, Norway

[5]Centre for Bioinformatics, Department of Pharmacy, University of Oslo, Oslo, Norway

[6]Division of Gynecologic Oncology, Department of Obstetrics, Gynecology and Reproductive Biology, Brigham and Women's Hospital, Boston, MA, United States

[7]Harvard Medical School, Boston, MA, United States.
[8]Department of Radiation Oncology, Dana-Farber Cancer Institute, Boston, MA, United States

[9]Department of Epidemiology and Biostatistics, School of Public Health, Imperial College London, London, UK

*** Correspondence:**

Konrad Stawiski, M.D., Ph.D.
Department of Biostatistics and Translational Medicine
Medical University of Lodz
Mazowiecka 15, 92-215 Lodz, Poland
+48 42 42 272 53 85
konrad.stawiski@umed.lodz.pl

Renée Turzanski Fortner, Ph.D.
Cancer Registry of Norway
Norwegian Institute of Public Health
Postbox 5313 Majorstuen
0304 Oslo, Norway
+47 22 92 88 02
rtfo@kreftregisteret.no

Supplementary Figures and Tables

Supplementary Figures

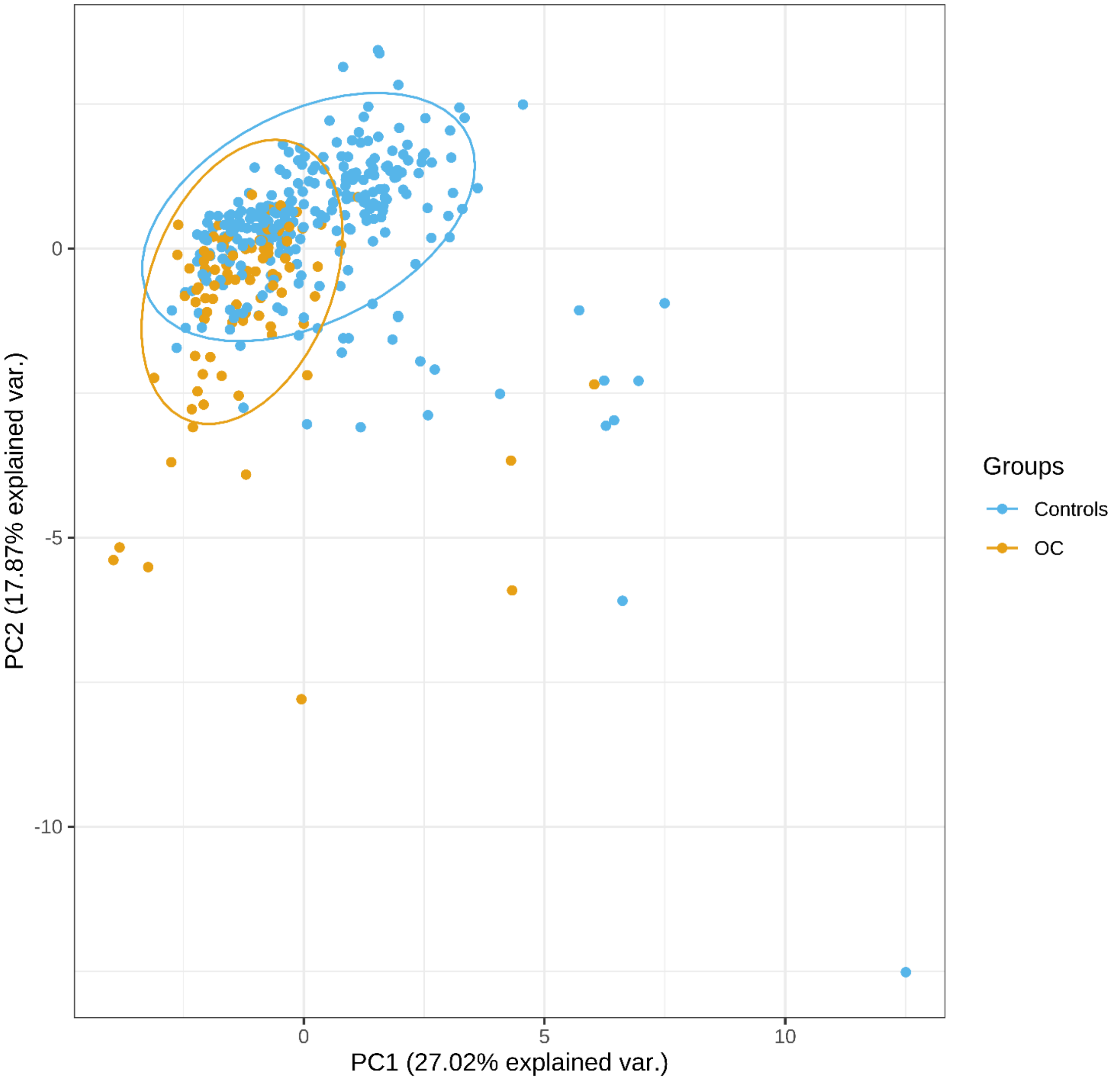

Supplemental Figure 1. The principal component analysis showed moderate differences due to batch effect in RNA profiles between ovarian cancer cases and controls in the JanusRNA study.

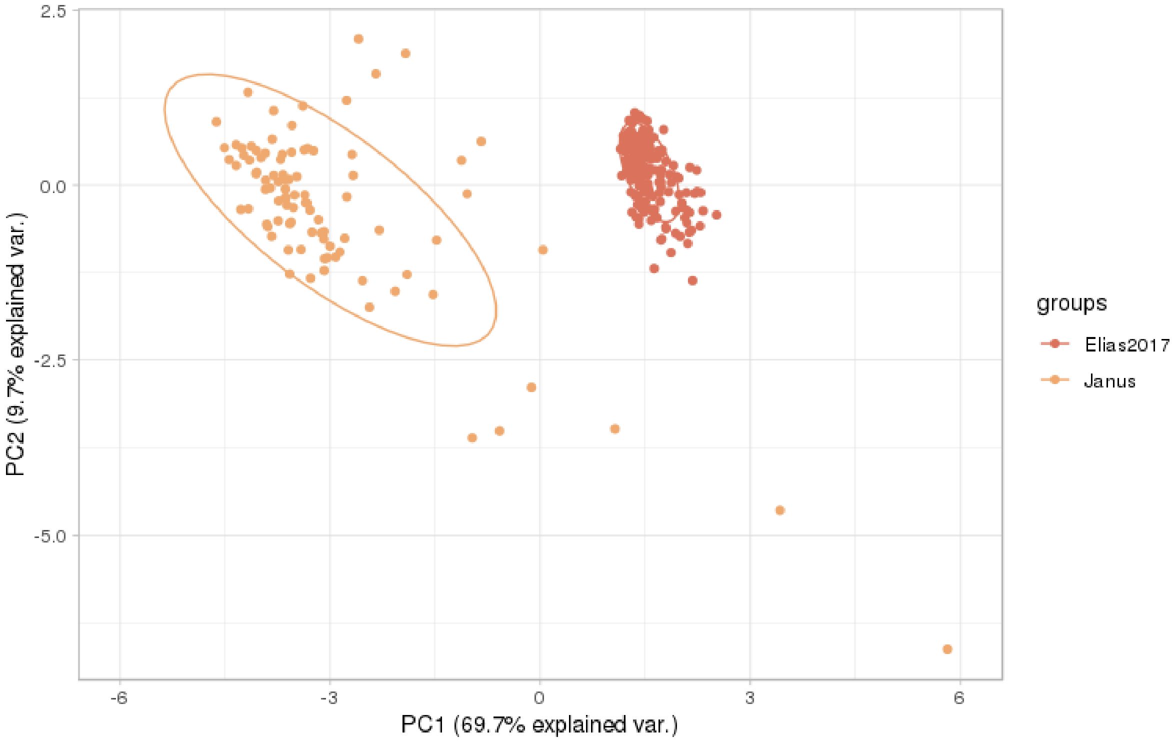

Supplemental Figure 2. The initial principal component analysis showed significant differences in RNA profiles by study when comparing the data from Elias 2017 and the JanusRNA study.

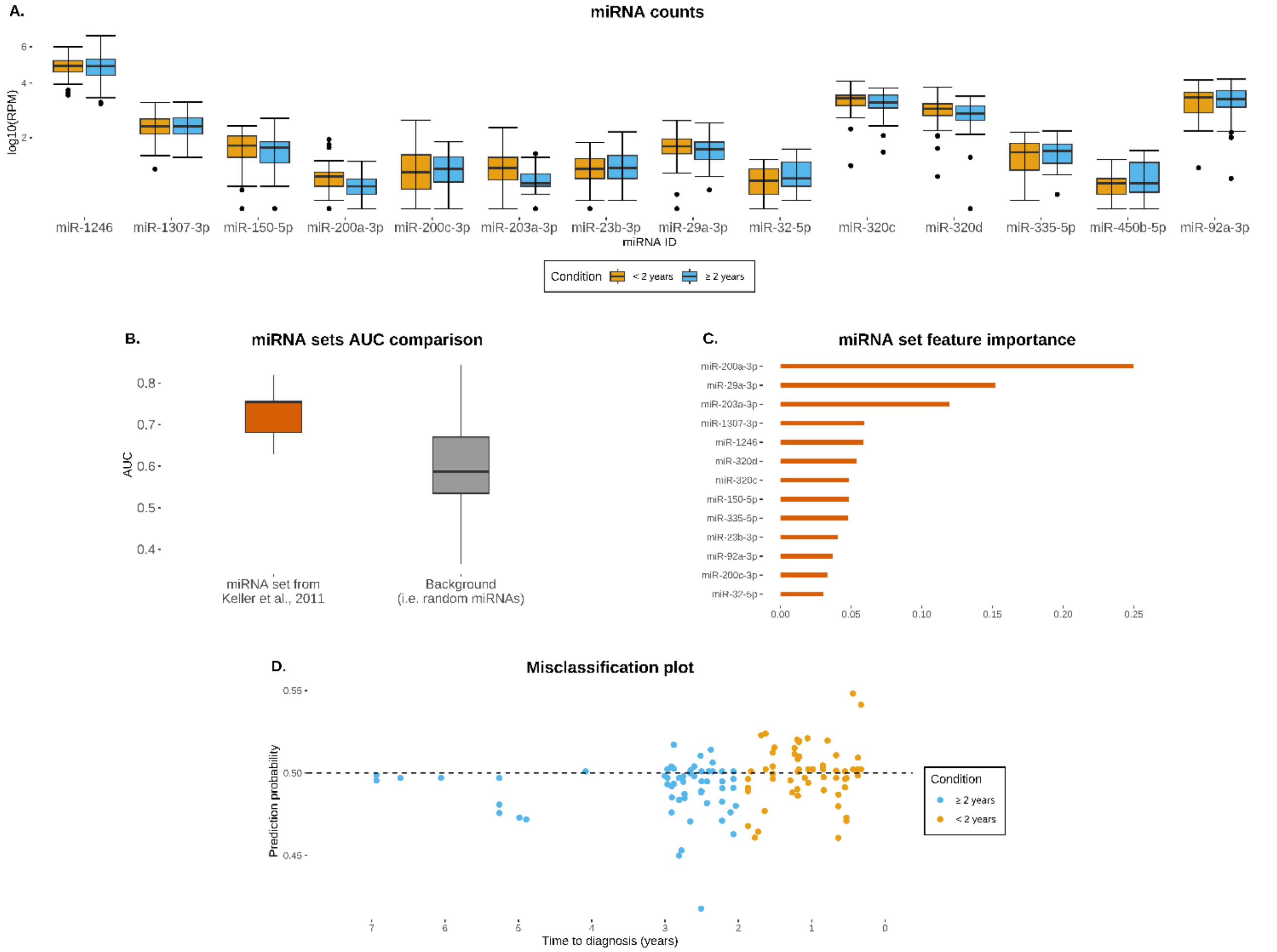

Supplemental Figure 3. Evaluation of miRNA set from dataset described in Keller et al. (2017) in JanusRNA ovarian cancer case set. **(a)** miRNA counts for the evaluated panel, by time between blood collection and diagnosis (<2 years, ≥2 to 7 years; microRNAs log10 transformed for visualization and zero values omitted from the plot); **(b)** Boxplots of model performance for the primary evaluated miRNA signature and random miRNA set; **(c)** Feature importance for each of the included miRNAs **(d)** Misclassification plot of the test samples based on the miRNA signature, y axis represents prediction probability for a given observation to have been sampled within 2 years prior to diagnosis.

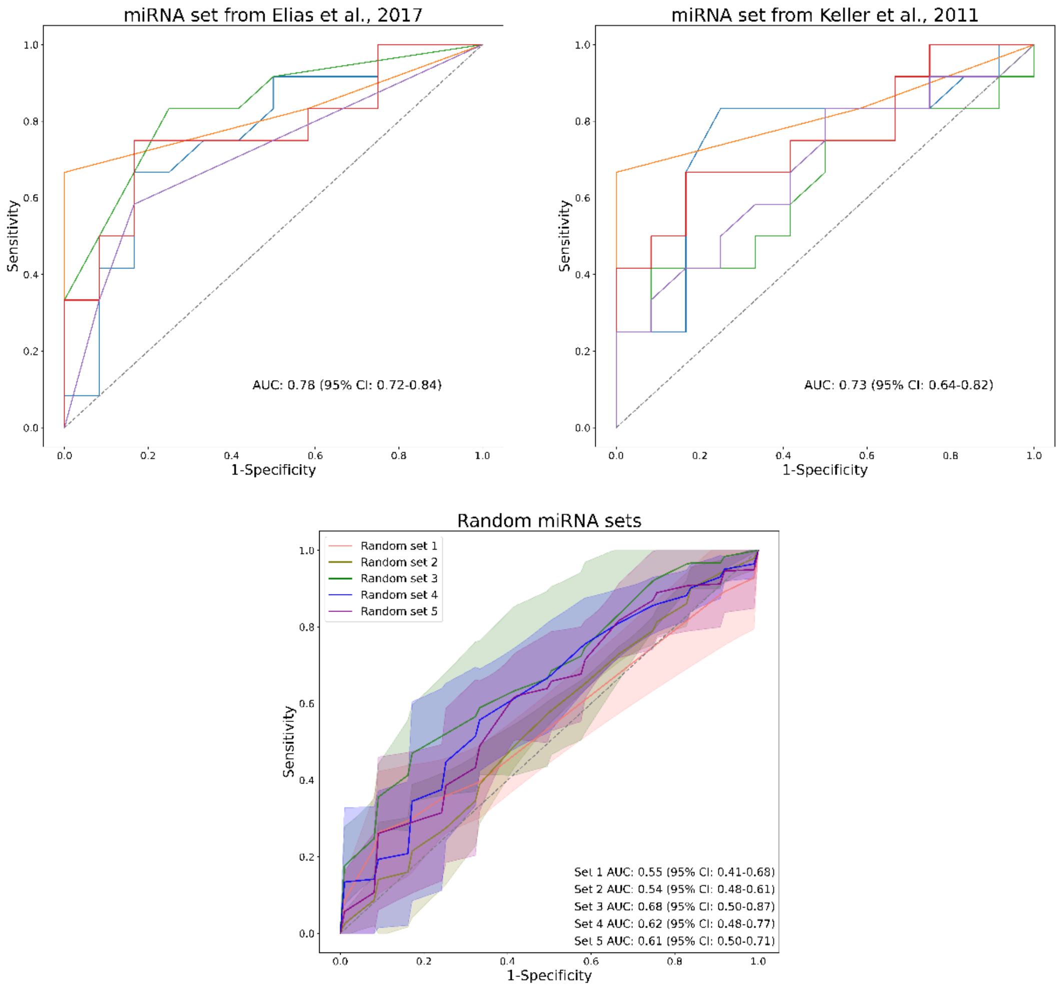

Supplemental Figure 4. ROC curves for the XGBoost model on the evaluated miRNA sets. Each curve represents one of the five training/test split performed. The average AUC and relative 95% confidence intervals are also shown for each of the miRNA sets. In the random miRNA sets figure, each curve represents the average of each of the five training/test split repetitions curve and its ± one standard deviation from the mean.

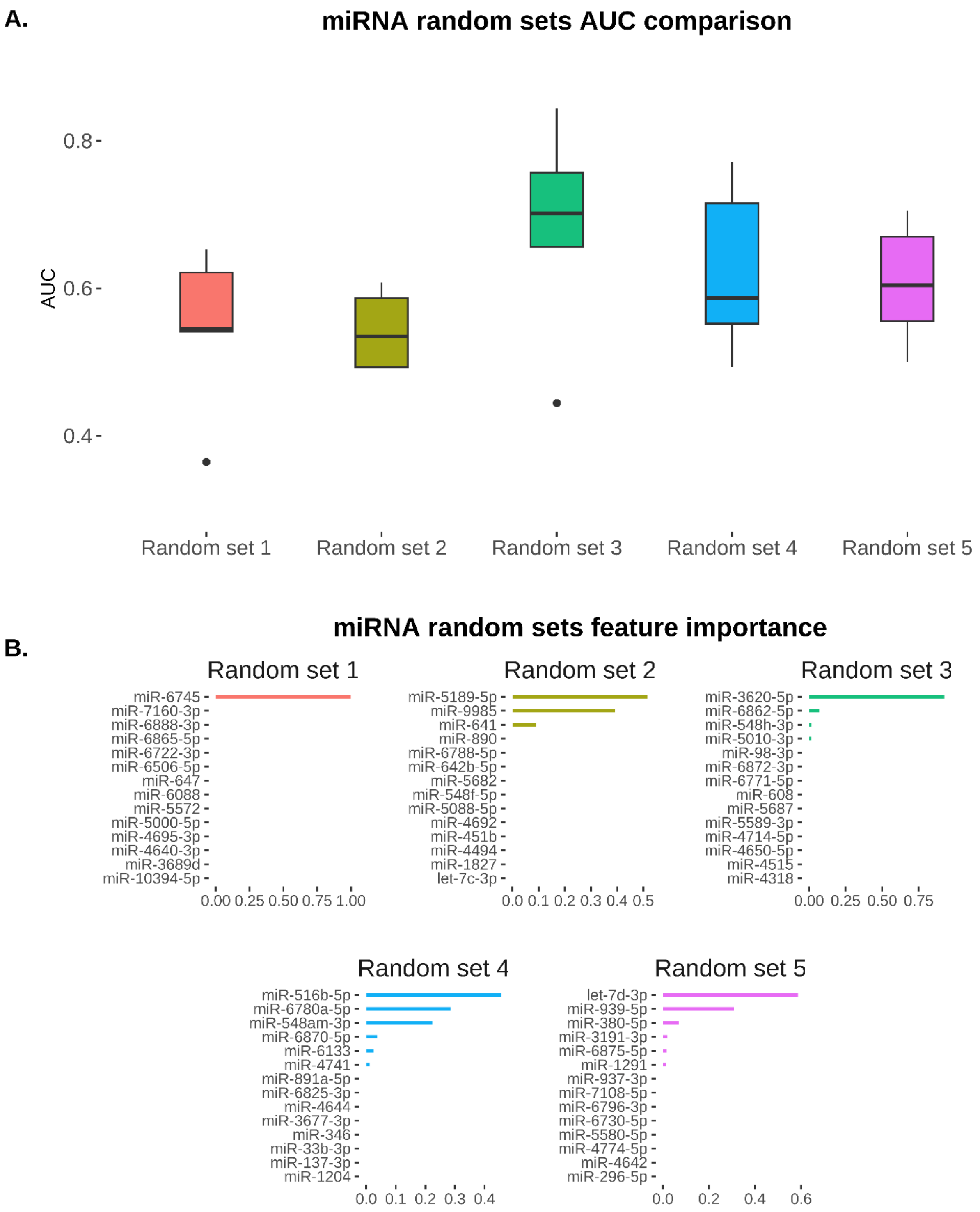

Supplemental Figure 5. Boxplots of model performance for the five sets randomly selected miRNAs (14 random miRNAs for each set).

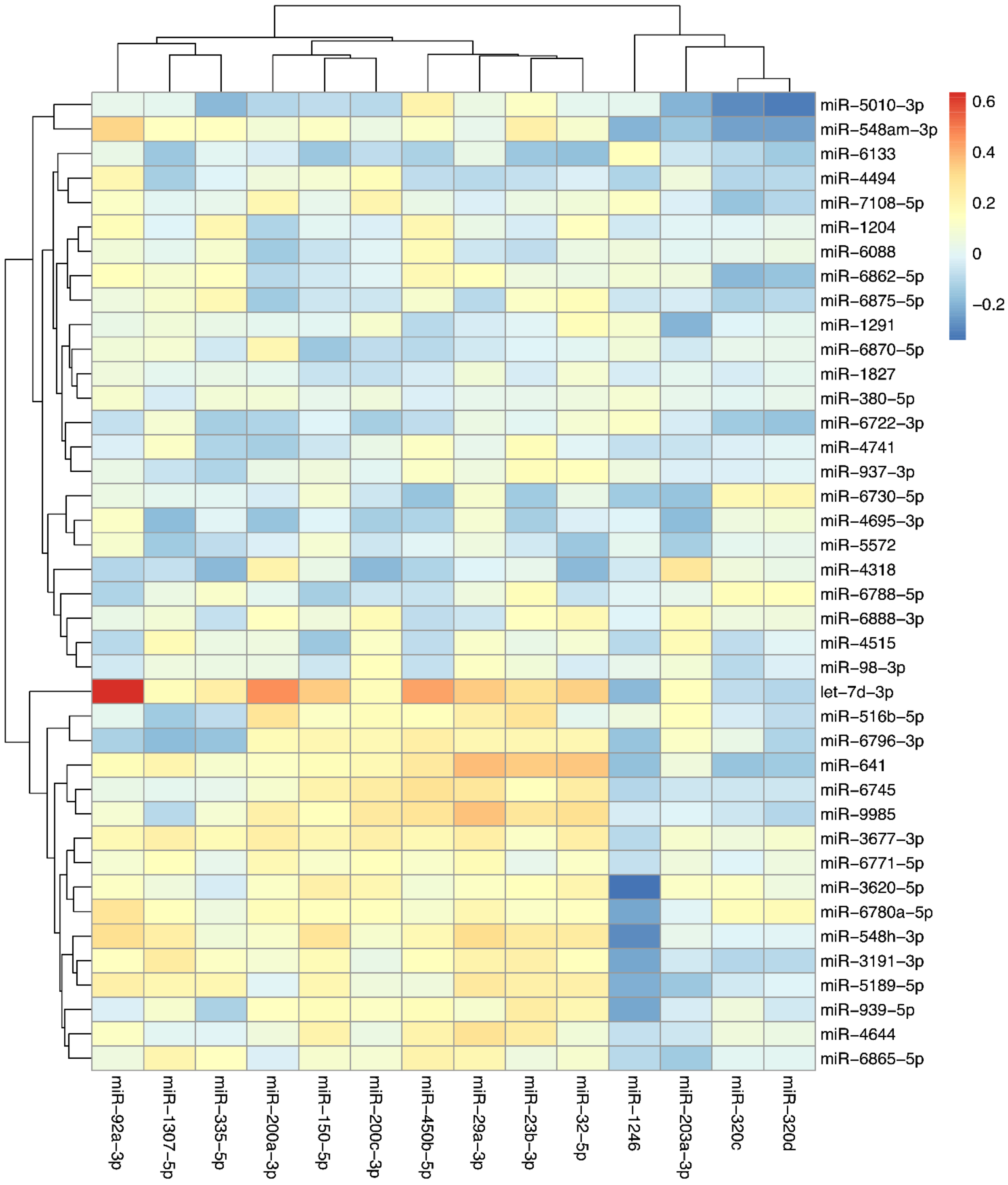

Supplemental Figure 6. Spearman correlation heatmap between the miRNA set from Elias et al. (2017) and the randomly selected miRNA with non-zero counts modelled in Supplemental Figure 4.

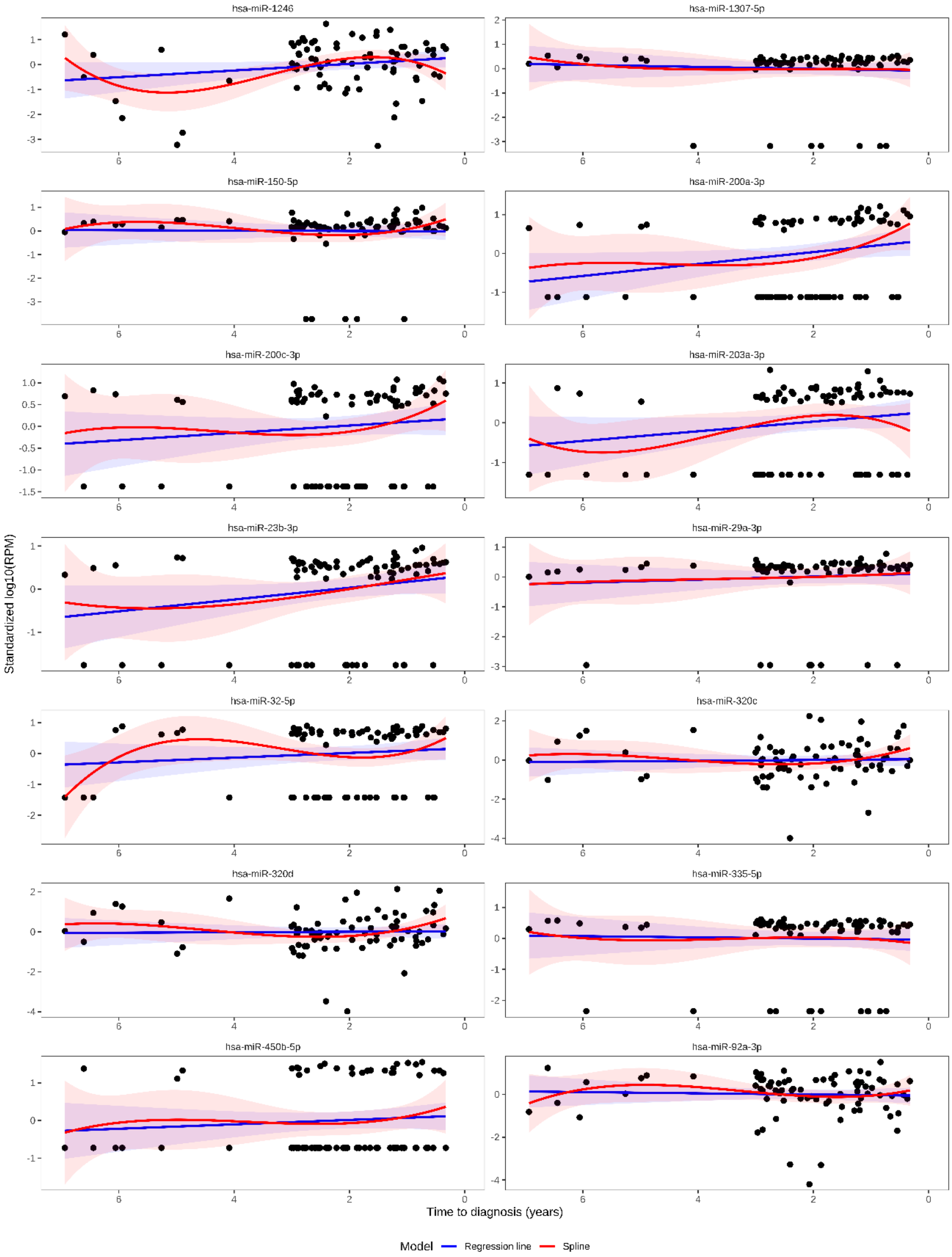

Supplemental Figure 7. Standardized $\log_{10}$-transformed RPM miRNA counts (z-score of $\log_{10}$ transformed values) dynamics from dataset described in Elias et al. (2017) in JanusRNA ovarian cancer case set.
